# Negative pressure wound therapy for the management of deep brain stimulation‐related surgical site infections: A retrospective case series

**DOI:** 10.1002/ibra.12178

**Published:** 2024-09-23

**Authors:** Si‐Yu Yan, Yi‐Fan Liu, Yi‐Cheng Zhou, Yuan Gao, Yang Wu, Hao Deng, Cheng‐Hao Yang, Jun‐Wen Guan, Wei Wang, Rui Tian

**Affiliations:** ^1^ Department of Neurosurgery, West China Hospital Sichuan University Chengdu Sichuan China; ^2^ West China School of Medicine Sichuan University Chengdu Sichuan China; ^3^ Department of Neurosurgery Zigong Fourth People's Hospital Zigong Sichuan China

**Keywords:** deep brain stimulation, negative pressure wound therapy, surgical site infection

## Abstract

The management of deep brain stimulation (DBS)‐related surgical site infection (SSI) is challenging. This article aimed to report the efficacy of negative pressure wound therapy (NPWT) in treating DBS‐related SSI while preserving all DBS devices. As a retrospective case series in a single center, localized DBS‐related SSI was treated with complete debridement and NPWT, with preserving all DBS devices. Successful infection control was defined as no clinical or microbiological evidence of recurrent infection 3 months after NPWT. Five patients (three females, two males, median age: 64 years) received NPWT for their DBS‐related SSI. The infection was located in the chest, parietal, and retroauricular areas. Only one patient had the extension wires removed due to the heavy contamination, while no DBS devices were removed in the other patients. All patients showed successful infection control without any remarkable side effects 3 months after debridement and NPWT. These findings suggest that NPWT may effectively promote wound healing with a high probability of preserving all DBS devices in DBS‐related SSI.

## Graphical abstract

The schema of our procedure is to use negative pressure wound therapy (NPWT) for the deep brain stimulation‐related surgical site infection (SSI). It shows that after NPWT, patients could achieve successful infection control without any remarkable side effects. In conclusion, NPWT may effectively promote wound healing with a high probability of preserving all deep brain stimulation (DBS) devices in DBS‐related SSI.

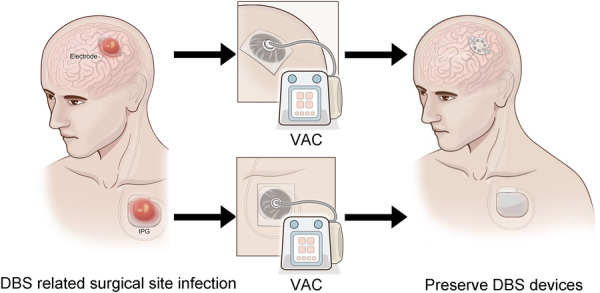

## 1 INTRODUCTION

Deep brain stimulation (DBS) is an efficient therapy that has been widely utilized for the management of various movement disorders, such as Parkinson's disease and essential tremor.^1^ DBS devices typically consist of an intracranial electrode, a subcutaneous extension wire, and an implantable pulse generator (IPG). Although DBS is a well‐established technique, DBS‐related surgical site infection (SSI) remains a serious complication, with an incidence of around 5.0%.^2^ Patients under immunocompromised conditions are more susceptible to DBS‐related SSI.

The conventional approach for managing DBS‐related SSI is complete hardware removal combined with intravenously administered antibiotics.^3^, ^4^ After the infection is completely controlled, reimplantation of new DBS hardware devices is necessary to treat primary disorders, which imposes additional burdens on patients. Some studies investigated the effectiveness of partial hardware removal in patients with localized infections to preserve the intracranial electrode or the IPG. However, the success rate was relatively low.^5^, ^6^

Negative pressure wound therapy (NPWT) is widely used to promote wound healing.^7^, ^8^ It has been found effective in treating complicated and nonhealing wounds including managing closed incisions to prevent SSI in some studies.^9^, ^10^, ^11^, ^12^, ^13^ However, no previous studies investigated the application of NPWT for managing DBS‐related SSI.

This study was designed to explore the therapeutic effect of NPWT for patients with SSI after DBS in a single center.

## 2 MATERIALS AND METHODS

This case series reported in line with the PROCESS Guideline.^14^

We included patients with DBS‐related SSI who were treated with NPWT and were followed up for at least 3 months at our hospital from November 2021 to October 2022. Demographical characteristics, treatment history, wound characteristics, and laboratory results were reviewed from the medical records. V.A.C. ULTA Negative Pressure Wound Therapy System (KCI Medical China Co., Ltd) was used. Successful infection control was defined as the absence of clinical or microbiological evidence of recurrent infection 3 months after NPWT.

**Figure 1:**
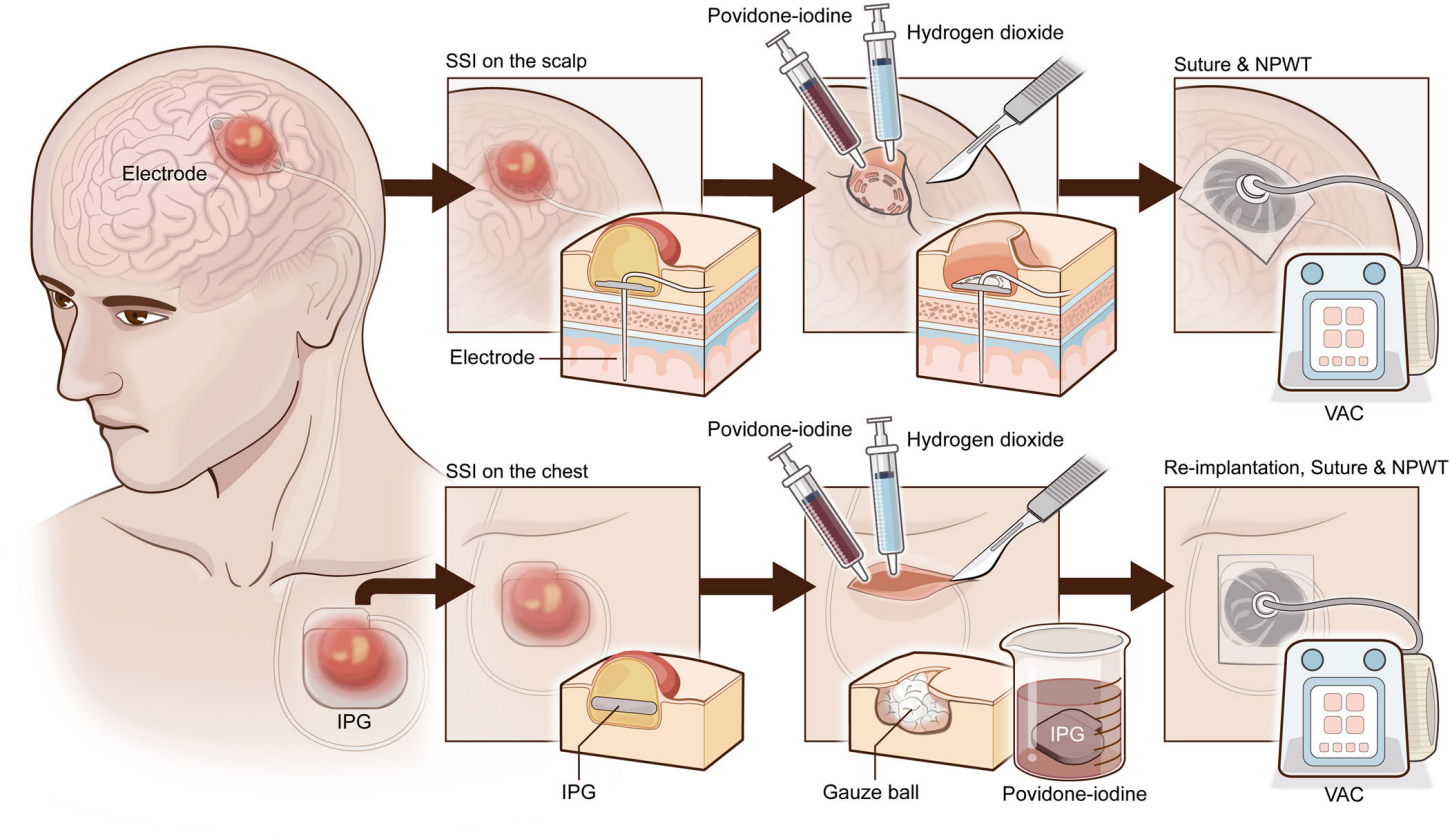
The schema of our procedure to use negative pressure wound therapy for the deep brain stimulation‐related surgical site infection. IPG, implantable pulse generator; NPWT, pressure wound therapy; SSI, surgical site infection; VAC, vacuum‐assisted closure. [Color figure can be viewed at wileyonlinelibrary.com]

For patients with DBS‐related SSI on the scalp, we prepared the entire scalp skin and made an incision following the previous surgical cut. Then, all dead, damaged, and infected tissues were removed carefully until fresh granulation tissue was exposed. The extension wires were removed if they were heavily contaminated or firmly attached to the infected tissue, preserved otherwise. Furthermore, the wounds were completely soaked with hydrogen dioxide solution, povidone‐iodine solution, or normal saline solution, three times each, for at least 1 min each time. After debridement, the wounds were sutured and closed, especially with the epicranial aponeurosis tightly sutured. An NPWT dressing was then applied to the wounds with continuous pressure of 100 mmHg (Figure 1).

For patients with infections presented on the chest, similar procedures were performed. After making the incision, the IPG was carefully explanted and soaked in povidone‐iodine. The IPG sheath was then thoroughly debrided, and gauze balls soaked with hydrogen dioxide or povidone‐iodine solution were stuffed into the sheath three times each, for at least 1 min each time. The sheath was then irrigated with normal saline solution. After debridement, the IPG was reimplanted into the same sheath and reconnected to the extension wires. After suturing, NPWT was applied to the wounds.

After operation, the patients received standard care. Antibiotics were intravenously administered until the termination of NPWT. Cefazolin was typically used as the empiric therapy. Then antibiotic regimens were adjusted according to the results of microbiological tests of the removed infected tissue. NPWT was discontinued when there was no drainage fluid for 24 h.

## 3 RESULTS

Five patients with DBS‐related SSI who were treated with NPWT were included in the study (Table 1). Among them, three were females and two were males, with a median age of 64 years. Four patients were diagnosed with Parkinson's disease, and one was diagnosed with dystonia. Two patients had diabetes and hypertension. The median duration between infection and the last DBS‐related surgery was 8 years (ranging from 1 to 15 years). The infection locations included chest, parietal, and retroauricular areas, and the most common symptoms were erythema, purulence, or erosion.

**Table 1:** Patients' information.

No.	Age (years)/gender	Diagnosis	Infection location	Presenting symptom	Time to infection (years)	Risk factor	Removal of DBS devices	NPWT draining time (days)	Antibiotics	Successful infection control
1	66/M	Parkinson's disease	Right parietal	Erythema, erosion	15	Diabetes, hypertension	Extension wires	6	Cefoxitin 1 g q8h 6 days	Yes
2	22/F	Dystonia	Right parietal	Erythema, purulence, erosion	5	No	No	5	Cefazolin 1 g q12h 6 days	Yes
3	64/F	Parkinson's disease	Right parietal, retro auricular	Erythema, erosion	14	No	No	6	Cefazolin 1 g q12h 4 days	Yes
4	58/F	Parkinson's disease	Right parietal	Erythema, purulence, erosion	1	No	No	4	Cefazolin 1 g q8h 5 days	Yes
5	66/M	Parkinson's disease	Right chest	Erythema, purulence, erosion	8	Diabetes, hypertension	No	22	Cefazolin 1 g q8h 5 days (sequentially) Levofloxacin 0.5 g qd 10 days	Yes

Abbreviations: DBS, deep brain stimulation; F, female; M, male; NPWT, pressure wound therapy; q, quaque; qd, quaque die.

**Figure 2:**
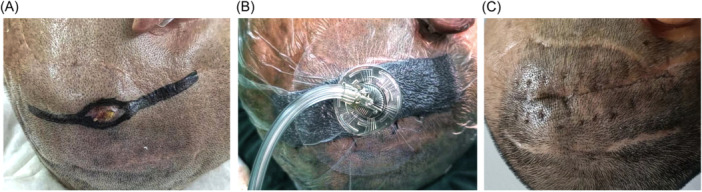
The infection site of Patient No. 1. (A) Erythema, erosion, and exposure of the extension wire were found on the right parietal area. (B) After complete debridement and removal of the extension wires, negative pressure wound therapy was performed. (C) The infection was controlled successfully, and the wound recovered well. [Color figure can be viewed at wileyonlinelibrary.com]

All patients received complete debridement of the infected area followed by NPWT (Figure 2). Four patients received NPWT for 4–6 days postoperatively, while one patient who had a localized infection at the IPG sheath received NPWT for 22 days. In Patient No. 1, the extension wires were removed because of the heavy contamination, while in other patients, no subassemblies were removed.

Three months after NPWT, all patients showed good wound healing with no clinical or microbiological evidence of recurrent infection. No side effects of the application of NPWT were reported. Especially, no leakage of cerebrospinal fluid was found.

## 4 DISCUSSION

The management of DBS‐related SSI is a significant challenge for clinicians. Although complete hardware removal is the standard method,^3^ some studies explored the option of partial removal of DBS devices. Sillay et al.^5^ treated 14 patients with localized SSI after DBS by partially removing the IPG and extension wire and administering intravenous antibiotics. Nine patients (64%) recovered and received further reoperations. They also attempted wound washout without hardware removal in one patient but failed.^5^ Li et al.^6^ developed an IPG‐sparing algorithm for managing patients with infection occurring in the frontal or postauricular area. They reported an IPG preservation rate of 70%.

To our knowledge, no studies explored the efficacy of NPWT for DBS‐related SSI to preserve DBS devices. NPWT can cover the entire wound to provide subatmospheric pressure, which has shown to increase blood flow, reduce edema, stimulate angiogenesis, and induce collagenation.^15^, ^16^ Besides, NPWT can draw the interstitial fluid from the wound and eliminate inflammatory factors and bacterial contamination which may impair wound healing.^16^, ^17^ Several meta‐analyses of randomized trials^7^, ^18^ have demonstrated that, compared with standard care, NPWT can reduce wound size, shorten wound healing time, and decrease hospital stay length.

The potential concern of cerebrospinal fluid leakage was a primary consideration in the application of NPWT. However, based on our case series, all patients demonstrated successful recovery, and no instances of cerebrospinal fluid leakage were observed. Additionally, Pujji et al.^19^ reported a case in which NPWT was effectively used over the dura to treat an infected full‐thickness scalp burn. These findings lead us to believe that NPWT can be cautiously used. Nevertheless, further prospective studies are needed to evaluate the safety of NPWT in neurosurgery.

Our case series showed that the application of NPWT is a promising method to treat DBS‐related SSI. This case series provides Class IV evidence that NPWT may be an effective strategy to treat DBS‐related SSI while preserving DBS devices. To our knowledge, this is the first study to highlight potential application of NPWT for the management of DBS‐related SSI.

However, this study had several limitations. First, this was a retrospective study with a small sample size, which may lead to bias and confounding factors. Second, as a case series report, there was no control group. Thus, further prospective randomized control studies with larger sample sizes are needed to validate the efficacy of NPWT for DBS‐related SSI.

## 5 CONCLUSION

NPWT may effectively promote wound healing with a high probability of the preservation of all DBS devices in DBS‐related SSI.

## AUTHOR CONTRIBUTIONS

Si‐Yu Yan, Yi‐Fan Liu, and Yi‐Cheng Zhou designed the study, acquired data, reviewed for consistency of data, analyzed data, drafted and revised the manuscript. Yuan Gao, Yang Wu, and Hao Deng reviewed for appropriateness of data. Cheng‐Hao Yang reviewed for appropriateness of data and revised the manuscript. Jun‐Wen Guan supervised the study and revised the manuscript. Wei Wang and Rui Tian designed the study, supervised the study, and revised the manuscript.

## CONFLICT OF INTEREST STATEMENT

The authors declare no conflict of interest.

## ETHICS STATEMENT

This study was approved by the ethics committee of West China Hospital, Sichuan University (2023615). All patients signed the informed consent.

## Data Availability

Anonymized data not published within this article will be made available by request from any qualified investigator.
